# Prognostic value of [^18^F]FDG- and PSMA-PET in patients evaluated for [^177^Lu]Lu-PSMA therapy of mCRPC

**DOI:** 10.1007/s00259-025-07198-y

**Published:** 2025-03-21

**Authors:** Tugce Telli, Leonor Lopes, Madeleine Karpinski, Kim M. Pabst, Viktor Grünwald, Kuangyu Shi, Boris Hadaschik, Claudia Kesch, Lale Umutlu, Ken Herrmann, Robert Seifert, Wolfgang P. Fendler

**Affiliations:** 1 Department of Nuclear Medicine, University Hospital Essen, Essen, Germany; 2 German Cancer Consortium (DKTK), Partner Site, Partnership Between DKFZ and University Hospital Essenaq , Essen, Germany; 3 West German Cancer (WTZ), Essen, Germany; 4 Department of Nuclear Medicine, University Hospital Bern, Bern, Switzerland; 5 Department of Urology, University Hospital Essen, Essen, Germany; 6 Department of Diagnostic and Interventional Radiology and Neuroradiology, University Hospital Essen, Essen, Germany

**Keywords:** FDG, PSMA, Prognostics, Biomarkers, Prostate cancer

## Abstract

**Purpose:**

To improve [^177^Lu]Lu-Prostate-specific membrane antigen therapy (LuPSMA) selection, this study investigates the prognostic value of PSMA and 2-[^18^F]fluoro-2-deoxy-D-glucose ([^18^F]FDG)-PET in metastatic castration-resistant prostate cancer (mCRPC) patients considered for LuPSMA therapy.

**Methods:**

We conducted a retrospective analysis in 152 mCRPC patients referred for LuPSMA therapy who underwent PSMA and [^18^F]FDG-PET/CT. Of these, 104 patients (68.4%) underwent LuPSMA therapy, while 48 (31.6%) received other standard of care (SOC). PET/CT analyses included visual assessment and semiquantitative measurements. Clinical and laboratory parameters were recorded. Overall survival (OS) and PSA response (decline > 50%) were primary and secondary endpoints, respectively.

**Results:**

Baseline [^18^F]FDG-derived total tumor volume was the only independent predictor of overall survival both in patients subsequently treated with LuPSMA (HR 1.28 [95%CI 1.02—1.61]; p = 0.03) or in those under other SOC (HR 1.61 [95%CI 1.02—2.56]; p = 0.04), respectively. In other SOC patients, additional independent predictors of OS were total lesion PSMA uptake (PSMA-TL; HR 1.14 [95%CI 1.03–1.26]; p = 0.01), [^18^F]FDG mean SUV (HR 20.88 [95%CI 1.2–364.74]; p = 0.04), and [^18^F]FDG total lesion glycolysis (HR 1.61 [95%CI 1.02–2.56]; p = 0.04). In LuPSMA patients, PSMA-PET SUVmean was a significant independent predictor of PSA decline ≥ 50% (OR 2.97 [95%CI 1.27–8.16]; p = 0.02).

**Conclusion:**

PSMA-PET and [^18^F]FDG-PET provide imaging biomarkers of outcome in candidates for LuPSMA. FDG-PET total tumor volume was an independent predictor of overall survival in candidates for LuPSMA therapy, irrespective of subsequent treatment decision. PSMA-PET SUVmean was associated with biochemical response to LuPSMA. Dual tracer imaging should further be assessed in prospective trials for mCRPC treatment guidance.

**Supplementary Information:**

The online version contains supplementary material available at 10.1007/s00259-025-07198-y.

## Introduction

Metastatic castration-resistant prostate cancer (mCRPC) is characterized by a high degree of heterogeneity, leading to variable inter- and intra-patient therapeutic responses. Therefore, personalized treatment is recommended for the advanced stage of the disease [1]. Several molecular treatment pathways have been established: Currently, Poly (ADP-Ribose) Polymerase inhibitors are recommended as monotherapy or in combination with different treatment modalities in patients with deleterious mutations in DNA-repair genes, whereas platinum therapy is typically recommended for patients with other molecular signatures including TP53 or PTEN [2]. However, molecular imaging-guided treatment decisions in mCRPC have not been established broadly yet.

One of the novel personalized approaches is [^177^Lu]Lu-Prostate-specific membrane antigen radioligand therapy (LuPSMA) for patients with PSMA-expressing metastases. Molecular imaging parameters, such as PSMA total tumor volume and average standardized uptake value (SUVmean) on PSMA-PET have been associated with overall survival and treatment response to LuPSMA therapy [3, 4].

[^18^F]FDG-PET, which provides biomarker metrics for tumor aggressiveness, was systematically used in the LuPSMA trial by Hofman et al. to identify patients with aggressive lesions exhibiting low PSMA expression and to exclude them from LuPSMA therapy [5]. In a substudy of the TheraP trial, Buteau et al. reported that patients with metabolic tumor volume on [^18^F]FDG-PET ≥ 200 mL had lower PSA response rates and shorter progression-free survival, irrespective of treatment with Cabazitaxel or LuPSMA therapy [3]. This suggests that [^18^F]FDG-PET, in conjunction with PSMA-PET, may be a valuable tool for treatment guidance or outcome prognostication. Different 2-[^18^F]fluoro-2-deoxy-D-glucose ([^18^F]FDG)-PET derived parameters, such as sum of SUVmax of FDG-positive lesions, tracer uptake heterogeneity, and FDG-positive progression were found to provide independent prognostic information in mCRPC [6–8].

Despite a growing number of mCRPC therapeutic options, robust prognostic tools to guide treatment pathways or therapy stratification are still lacking. We explore the prognostic significance of PSMA- and [^18^F]FDG-PET imaging parameters in mCRPC patients and candidates for LuPSMA therapy, which may help improve candidate selection and optimize treatment strategies for LuPSMA therapy in the future.

## Materials and methods

### Study design and patients

A total of 152 patients were analyzed retrospectively. Patients were referred for [^177^Lu]Lu-PSMA-617 and underwent dual PSMA-PET/CT and [^18^F]FDG PET/CT at the Nuclear Medicine Department of Essen University Hospital from July 2019 until March 2022 as part of routine clinical practice. [⁶⁸Ga]Ga-PSMA-11 or [^18^F]PSMA-1007 PET/CT were used for imaging and evaluation of LuPSMA-related inclusion criteria in analogy to the VISION trial. Most patients (98%) with clinically significant FDG-PSMA mismatch findings were excluded from LuPSMA therapy. Exceptions occurred due to interdisciplinary decisions and lack of alternative treatments. Following PET/CT, treatment decisions were made in an interdisciplinary tumor board meeting, where a team of specialists, including oncologists, urologists, radiologists, nuclear medicine specialists, and pathologists collaboratively reviewed the imaging findings and clinical context. This approach aims to minimize bias by incorporating multiple perspectives. Eligible were mCRPC patients with disease progression after previous treatment both with one or more approved androgen-receptor–pathway inhibitors and with either one or two taxane regimens, who have at least one PSMA-positive metastatic lesion and no PSMA-negative lesion [9]. Patients underwent either LuPSMA or other standard of care (SOC), including, other alfa/beta emitter radionuclide therapy ([^223^Ra]RaCl2, [^90^Y]-FAPI, etc.), cytotoxic therapy (cabazitaxel, carboplatin, etc.), anti-androgen therapy or androgen receptor pathway inhibitor or best support care, at the discretion of the multidisciplinary team. Patients were followed until 1st of December 2023 to assess overall survival. The study was conducted in accordance with the Declaration of Helsinki. The local ethics committee approved this retrospective study (Permission Numbers: 22–10,822-BO/23–11,654) and waived the need for study-specific consent.

### Imaging protocol

As part of clinical routine, [^18^F]FDG, [⁶⁸Ga]Ga-PSMA-11, or [^18^F]PSMA-1007 PET/CT image acquisitions were initiated 60, 60, or 90–120 min after intravenous injection, respectively. Images were acquired using either Siemens Biograph 128 mCT or Biograph 64 VISION 600. Only patients who underwent [^18^F]FDG- and PSMA-PET/CT within time intervals of less than four weeks (mean: 3.1 days; SD: 6.3 days) were included in this study.

### Imaging analyses

#### Visual assessment

Visual assessment was performed by one experienced nuclear medicine physician (TT) and consensus reading was employed by three experienced readers (TT, RS, WF) for those patients with inconclusive results. Pathologic findings on [^18^F]FDG- and PSMA-PET images were assessed for local disease (T), pelvic lymph node metastases (N), distant lymph node metastases (M1a), bone metastases (M1b) and visceral metastases (M1c), including number of lesions and subregions involved in accordance with the PROMISE molecular imaging TNM classification [10].

[^18^F]FDG and PSMA PET images were compared visually for intensity and number of lesions. PSMA negative, [^18^F]FDG-positive lesions were determined as lesions with PSMA uptake lower than liver uptake with intense [^18^F]FDG uptake higher than liver. In addition, clinically relevant mismatch was defined as 1) visceral metastases/soft tissue lesions with longest diameter of ≥ 10 mm; 2) lymph nodes with short axis diameter exceeding 15 mm; 3) ≥ 3 bone metastases with [^18^F]FDG uptake higher than liver and PSMA uptake lower than liver. A summary of visual assessment criteria is presented in Supplementary Table 1.

#### Semiquantitative analyses

A summary of the performed semiquantitative analyses is presented in Supplementary Table 1. For selection and segmentation of positive lesions, RECIP 1.0 criteria for PSMA PET/CT (threshold of greater than 3 for bone lesions and 4.3x(mean SUV_liver_ + standard deviation_liver_)/mean SUV_liver_ for other regions) [11, 12]. Semi-automated segmentation and volumetry were applied by one experienced reader (TT) as defined by RECIP 1.0 [11, 12]. PARS and MICIIS 1.0 segmentation programs were used for [^68^ Ga]Ga-PSMA-11 and [^18^F]PSMA-1007 PET analyses [13, 14]. For soft tissue lesions that cannot be separated automatically from the physiological uptake of surrounding tissue, manual/adjusted contouring was used. Total tumor volume, tumor volume in local disease (T), pelvic lymph node metastases (N), distant lymph node metastases (M1a), bone metastases (M1b) and visceral metastases (M1c) were calculated separately. The average of the SUVmax and SUVpeak of the tumor lesions in the whole body were calculated. [^18^F]FDG-PET tumor quantification, as well as mismatch tumor quantification (i.e. [^18^F]FDG-PET/CT-derived volume of metastases with low PSMA-expression) were calculated according to PERCIST, i.e. an SUV threshold > (1.5 × mean SUV_liver_ + 2 × standard deviation_liver_) was used as the threshold for selecting and segmenting FDG-positive lesions [15].

[^18^F]FDG and PSMA imaging parameters were defined by the investigators before regression analyses based on previous literature. The PSMA imaging parameters selected for further analysis were PSMA mean SUV (PSMA-SUVmean)—calculated as the mean SUV of all segmented tumor voxels, PSMA total tumor volume (PSMA-VOL), total lesion PSMA uptake (PSMA-TL)—calculated as PSMA-SUVmean multiplied by PSMA-VOL, and total number of PSMA-positive lesions. The [^18^F]FDG parameters selected were [^18^F]FDG mean SUV (FDG-SUVmean)—calculated similarly to PSMA-, [^18^F]FDG total tumor volume (FDG-VOL), total lesion glycolysis (FDG-TLG) and, for untreated patients, FDG-TLG mismatch and FDG-VOL mismatch.

### Other clinical and laboratory parameters

Date of initial diagnosis, previous therapy lines, initial Gleason and ISUP Scores, tumor markers (PSA, ALP, LDH), total blood count, liver and kidney functions, and performance status at the time of PET scans were recorded. LuPSMA therapy cycles and cumulative doses as well as laboratory results were also recorded per cycle.

### Outcome

Overall survival (OS) was the primary endpoint of the entire cohort. In addition, a PSA decline of more than 50% (7) was evaluated as a binary variable (0 – No decline ≥ 50%; 1 – Any decline ≥ 50% during LuPSMA therapy and first three months after end of therapy) in the patients who were treated with LuPSMA.

### Statistical analysis

Statistical analysis was performed using R 4.1.2 and Python 3.9.12 with the libraries Lifelines 0.27.7 and Scipy 1.7.3. The compliance of variables to normal distribution was determined by the Kolmogorov–Smirnov test and statistical tests were chosen accordingly. Descriptive data were presented as mean ± standard deviation for normally distributed parameters or median (interquartile range) for skewed parameters. Kaplan–Meier analysis was performed to estimate the median overall survival with 95% confidence interval (95% CI) and the survival curves of both patients treated and non-treated with LuPSMA. Differences in overall survival between treated and non-treated patients were determined by log-rank test. Cox proportional hazards model was used to examine the association of individual parameters with overall survival. Statistically significant parameters in predicting overall survival were included as covariates in a multivariate Cox regression. Hazard ratios (HR) and 95% CI as well as p-values are presented. A logistic regression model with PSA decline of more or less than 50% as response binary variable was used to estimate the odds ratio with 95% CI of individual parameters. Patients without PSA follow-up information were excluded from logistic regression analysis (n = 6). Correlations were assessed by Spearman’s test. Two-sided p-values less than 0.05 were considered statistically significant.

## Results

### Patient characteristics

Between July 2019 and March 2022, 152 patients were included. A group of 104 (68.4%) patients were eligible and received [^177^Lu]Lu-PSMA-617 radioligand therapy (LuPSMA group) and 48 (31.6%) patients recieved other standard of care treatment (SOC group). Within the LuPSMA group, 72 (69%) underwent baseline [⁶⁸Ga]Ga-PSMA-11, and 32 (31%) [^18^F]PSMA-1007 PET/CT scan. [^177^Lu]Lu-PSMA-617 was administered for a median of 3 cycles (IQR 2). Within the SOC patients, 35 (73%) were imaged with [⁶⁸Ga]Ga-PSMA-11 and 13 (27%) patients with [^18^F]PSMA-1007. Of the 104 patients in LuPSMA group, 2 (2%) had FDG-PSMA mismatch findings. Of the 48 SOC patients, 21 (43.8%) had FDG-PSMA mismatch. Other reasons against [^177^Lu]Lu-PSMA-617 were low PSMA expression without any FDG-PSMA mismatch (n = 3, 6.3%), low hematological reserve or low performance status (n = 5, 10.4%), kidney (n = 5, 10.4%) or liver (n = 1, 2%) impairments, whereas in 13 (27.1%) the reason of not receiving therapy was multifactorial. The median follow-up was 10.8 (IQR 12.2) months for LuPSMA patients and 5.6 (IQR 8.7) months for SOC patients. Of the 104 LuPSMA patients, 86 (82.7%) had died by the last follow-up. Details of patient characteristics are given in Table 1.

**Table 1:** Patient characteristics. Data are shown as median (IQR) or *n* (%). The *p* values of comparisons are shown. Either the Mann–Whitney U test (for continuous variables) or exact Fisher test (for categorical variables) were used

Patient characteristics	LuPSMA	Other SOC	p value
Number of patients	104	48	
Age	71.5 (65.8–78.3)	71.5 (65.0–78.3)	0.897
Gleason Score	8 (8–9), n = 81	9 (8–9), n = 40	0.566
Time since mCRPC (years)	2.6 (1.9–4.2)	2.1 (1.1–6.1)	0.546
Blood Parameters			
Prostate-specific antigen	142.5 (40.7–504.8)	83.6 (28.5–293.5)	0.331
Lactate Dehydrogenase	269.5 (225.0–394.8)	548.0 (264.0–852.0)	**0.0007**
Creatinine	0.97 (0.8.−1.1)	0.84 (0.7–1.2)	0.123
Aspartate aminotransferase	29.0 (20.8–47.3)	33.0 (23.0–56.5)	0.192
Alkaline Phosphatase	158.5 (94.0–320.8)	132.5 (94.8–393.5)	0.845
Hemoglobin	11.4 (10.1–12.5)	11.1 (9.5–12.2)	0.682
Leukocytes	6.5 (5.5–8.0)	7.6 (5.7–8.9)	0.262
Platelets	250.0 (210.8–318.0)	262.0 (196.0–340.0)	0.795
Metastases location on baseline PSMA-PET			
Local lymph nodes (miN1 or N2)	66 (63%)	22 (46%)	0.052
Distant lymph nodes (miM1a)	69 (66%)	27 (56%)	0.278
Bone (miM1b)	100 (96%)	44 (92%)	0.262
Liver	16 (15%)	14 (29%)	0.078
Lung/pleura	19 (17%)	5 (13%)	0.243
Lines of prior therapy after mCRPC	3 (2–4)	3 (2–4)	0.344
History of previous therapies			
Abiraterone	85 (82%)	38 (79%)	0.825
Enzalutamide	79 (76%)	28 (58%)	**0.035**
Docetaxel	90 (87%)	38 (79%)	0.338
Cabazitaxel	37 (36%)	9 (19%)	**0.038**
PSMA-PET imaging agent			
[⁶⁸Ga]Ga-PSMA-11	72 (69%)	35 (73%)	0.705
[^18^F]-PSMA-1007	32 (31%)	13 (27%)	0.705
LuPSMA therapy			
Number of LuPSMA cycles	3 (2–4)	-	-
Time between PSMA PET and LuPSMA [days]	28.5 (16.0–56.0)	-	-
Cumulated activity [GBq]	19.4 (13.4–29.9)	-	-
Deaths	86 (83%)	44 (92%)	0.214
PSA decline > 50%	43 (44%), n = 98	-	**-**

In the other SOC group, 11 (22.9%) received other alpha/beta emitter radionuclide therapy (n = 10 (90.9%) [^223^Ra]RaCl2, n = 1 (9.1%)[^90^Y]-FAPI), 9 patients (18.8%) received best supportive care, 8 (16.7%) received cytotoxic therapy (cabazitaxel, carboplatin etc.), and 2 (4.1%) received anti-androgen or androgen receptor pathway inhibitor therapy, whereas in 18 patients (37.5%) the therapy management was not available. In the SOC group, 44 (91.7%) of 48 had died by the last follow-up.

### Overall survival

#### Kaplan–Meier analysis

**Fig. 1:**
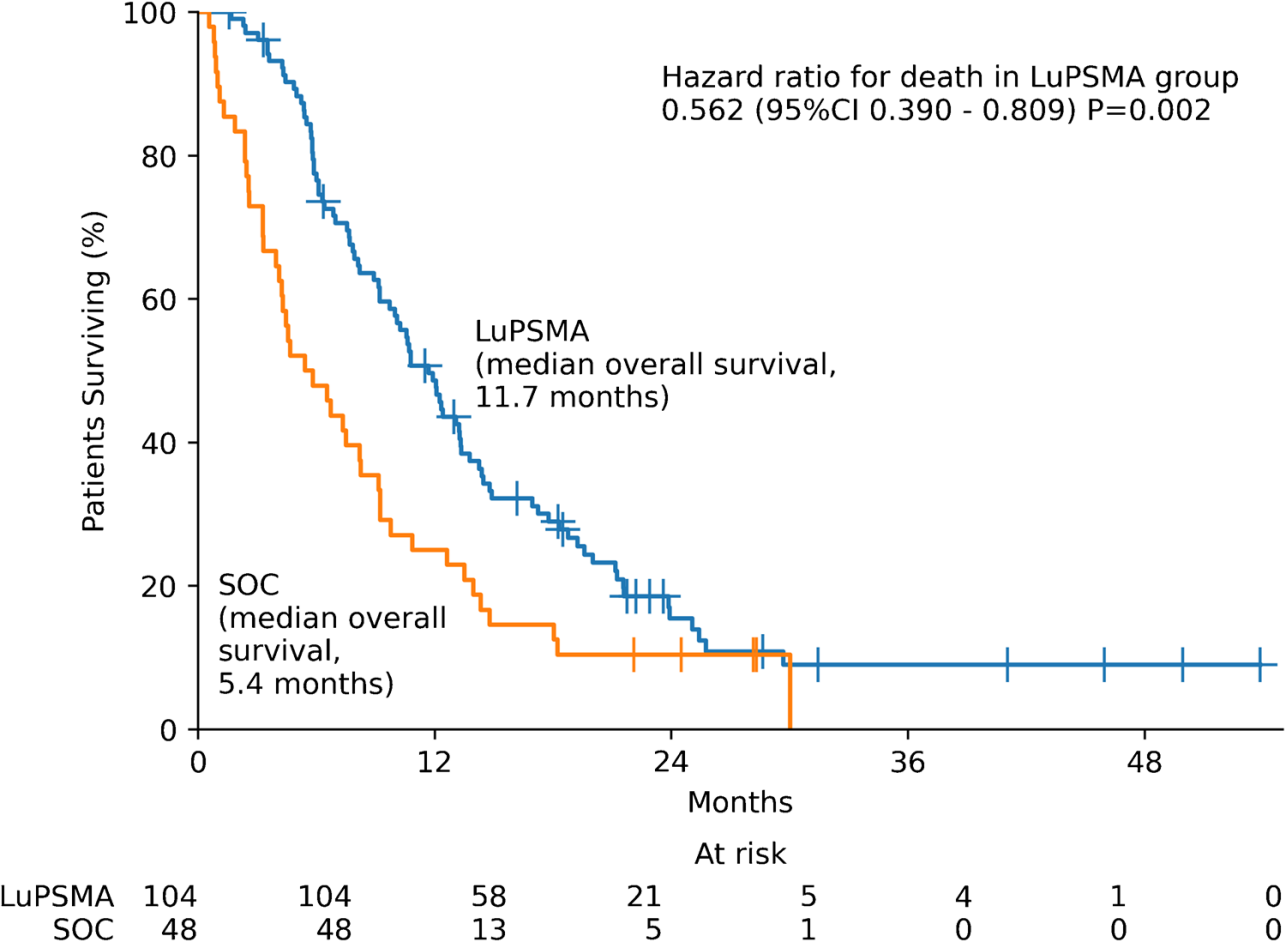
Overall Survival for LuPSMA versus other SOC patients. Median overall survival is shown for both LuPSMA and SOC groups. The *p*-value of the log-rank test for the comparison of overall survival between both groups is shown. Hazard ratio for death in LuPSMA in comparison to SOC was determined by Cox regression. Number of patients at risk in each time point is given below the plot

Figure 1 shows survival curves for the LuPSMA and other SOC groups. Median overall survival was significantly longer in the LuPSMA group versus the other SOC group (11.7 vs. 5.4 months, p = 0.002). The estimated 12-month overall survival was 47.7% (95% CI 37.7—57.0) for LuPSMA patients and 22.9% (95% CI 12.3—35.5) for other SOC patients.

Patients with FDG-PSMA mismatch findings had a shorter OS than patients without, regardless of the therapy received (4.5 months [95%CI 3.8–5.1] vs. 10.6 months [8.5–12.7], p = 0.01), respectively.

#### Univariate cox proportional hazards model

**Fig. 2:**
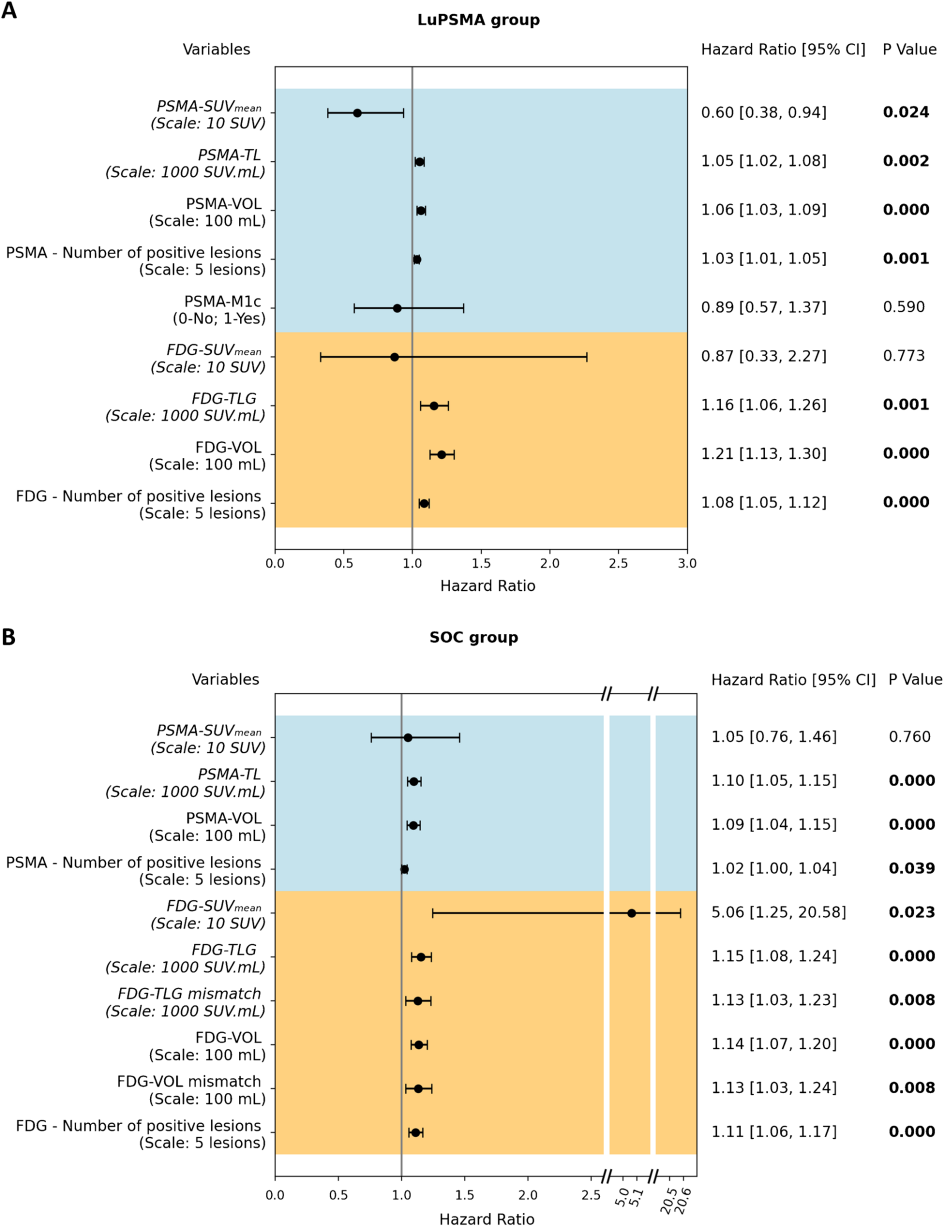
Forest plots of univariate cox proportional hazards model in **A** patients treated with LuPSMA and **B** other SOC. Hazard ratios, 95% confidence intervals (95% CI) as well as p-values of each univariate cox model are presented

In LuPSMA patients, all PET parameters except PSMA-M1c and FDG-SUVmean were significant prognostic factors of overall survival (Fig. 2A). PSMA-SUVmean (HR 0.60 [95%CI 0.38—0.93]; p = 0.024) was a significant protective factor and PSMA-TL (HR 1.05 [95%CI 1.02—1.08]; p = 0.002), PSMA-VOL (HR 1.06 [95%CI 1.03—1.09]; p < 0.001), number of PSMA-positive lesions (HR 1.03 [95%CI 1.01—1.05]; p = 0.001) were prognostic of worse outcome. Regarding [^18^F]FDG-PET parameters, FDG-TLG (HR 1.16 [95%CI 1.06—1.26]; p = 0.001), FDG-VOL (HR 1.21 [95%CI 1.13—1.30]; p < 0.001) and number of FDG-positive lesions (HR 1.08 [95%CI 1.05—1.12]; p < 0.001) were prognostic of worse outcome.

In other SOC patients, PSMA-TL (HR 1.10 [95%CI 1.05—1.15]; p < 0.001), PSMA-VOL (HR 1.09 [95%CI 1.04—1.15]; p < 0.001), number of PSMA-positive lesions (HR 1.03 [95%CI 1.01—1.05]; p = 0.012) were significantly prognostic of worse outcome (Fig. 2B). For [^18^F]FDG-PET, FDG-SUVmean (HR 5.06 [95%CI 1.25—20.58]; p = 0.023), FDG-TLG (HR 1.15 [95%CI 1.08—1.23]; p < 0.001), FDG-TLG mismatch (HR 1.13 [95%CI 1.03—1.23]; p = 0.008), FDG-VOL (HR 1.14 [95%CI 1.08—1.20]; p < 0.001), FDG-VOL mismatch (HR 1.13 [95%CI 1.03—1.24]; p = 0.008), and number of FDG-positive lesions (HR 1.11 [95%CI 1.06—1.17]; p < 0.001) were prognostic of worse outcome.

Univariate Cox proportion hazards model results for several other parameters are shown in Supplementary Tables 2 and 3, for LuPSMA and other SOC groups respectively (hazard ratio, 95% confidence interval, and p value). As some patients had baseline [⁶⁸Ga]Ga-PSMA-11 (n = 107) and others [^18^F]PSMA-1007 (n = 45), we also assessed univariate cox results separated by radiotracer in Supplementary Figs. 1 and 2. Interestingly, PSMA SUVmean on [^18^F]PSMA-1007 was not a statistically significant prognosticator of OS in the LuPSMA group, whereas PSMA-TL was (Supplementary Fig. 2).

#### Multivariate cox proportional hazards model

**Fig. 3:**
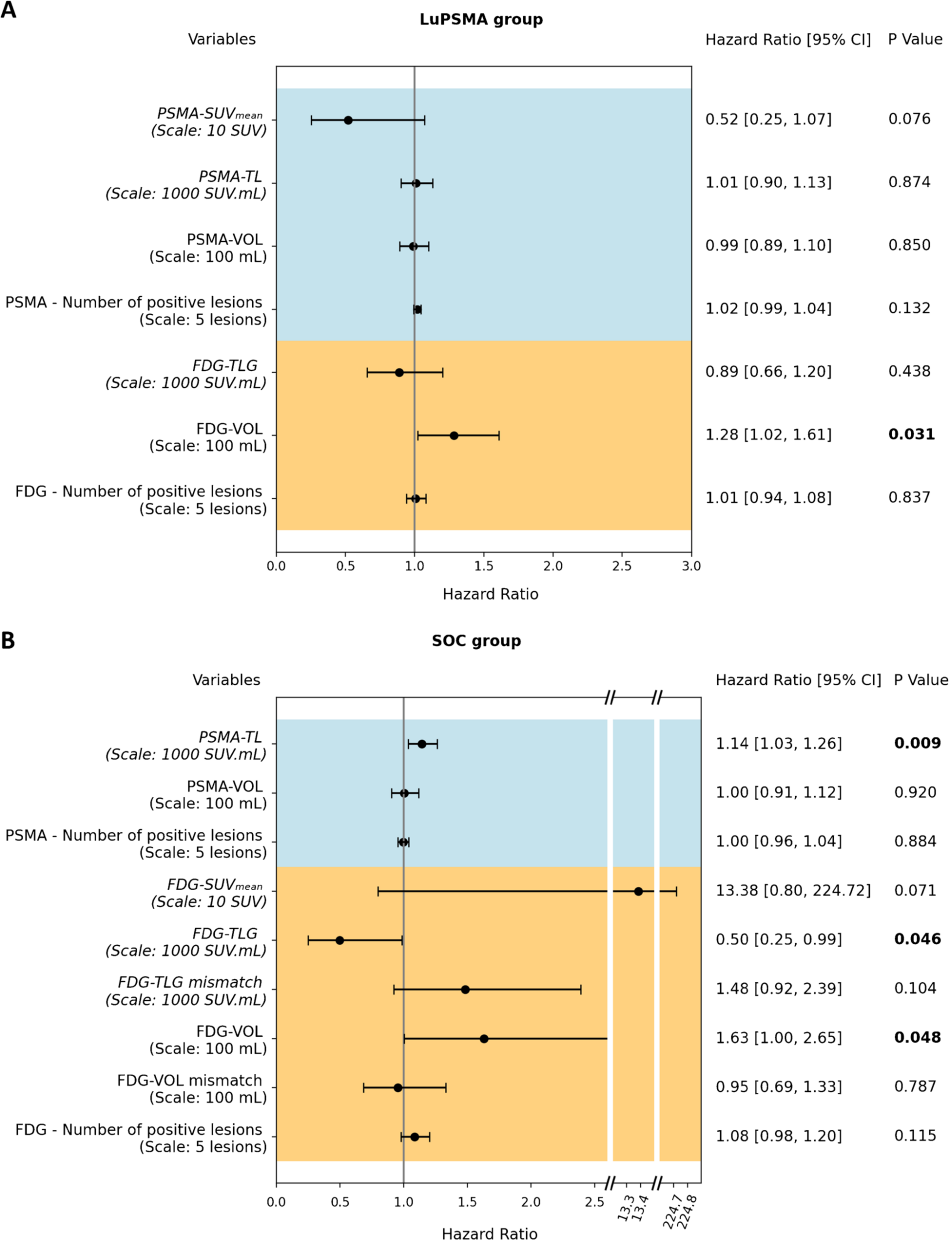
Forest plots of multivariable cox proportional hazards model in **A** patients treated with LuPSMA and **B** other SOC. Hazard ratios, 95% confidence intervals (95% CI) as well as p-values of the multivariate cox model of all the variables are presented

Of previously selected parameters, significant ones were included in a multivariate Cox proportional hazard model. In this multivariate model and in patients treated with LuPSMA only FDG-VOL remained significant (HR 1.28 [95%CI 1.02—1.61]; p = 0.031) (Fig. 3).

In other SOC patients, PSMA-TL (HR 1.14 [95%CI 1.03—1.26]; p = 0.009) and FDG-VOL (HR 1.63 [95%CI 1.00—2.65]; p = 0.048) were prognostic for short survival. FDG-TLG (HR 0.50 [95%CI 0.25—0.99]; p = 0.046) was prognostic for longer overall survival (Fig. 3).

#### ***Overall survival by [***^***18***^***F]FDG total volume quartiles***

**Fig. 4:**
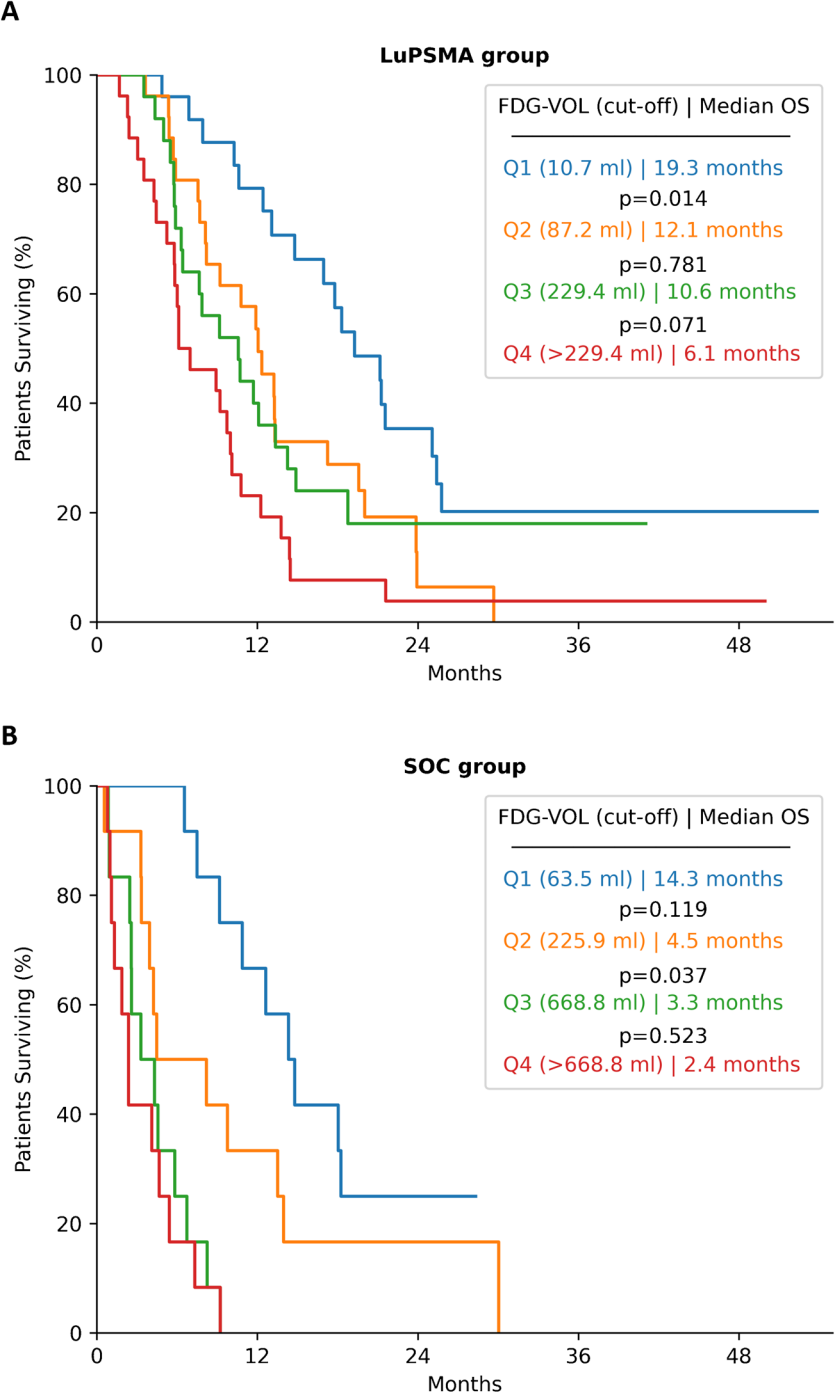
Overall survival curves stratified into[^18^F]FDG total tumor volume quartiles of **A** LuPSMA and **B** SOC groups. Patients were assigned to 4 quartiles according to FDG-VOL. Cut-off values of quartile partitions are shown in parenthesis. Median overall survival is shown for each quartile. The *p*-value of the log-rank test for pairwise comparison of overall survival between quartiles is shown

FDG-VOL was the only independent prognostic factor of overall survival in the LuPSMA and other SOC groups. We further explored this parameter: Kaplan–Meier plots of overall survival by FDG-VOL quartiles are shown in Fig. 4. The median overall survival in the LuPSMA group was 19.3 months in quartile 1, 12.1 in quartile 2, 10.6 months in quartile 3, and 7.0 months in quartile 4, respectively. In the other SOC group, the median overall survival was 14.3 months in quartile 1, 4.5 months in quartile 2, 3.3 months in quartile 3 and 2.4 months in quartile 4, respectively. Log-rank test showed significant differences between quartiles 1 and 2 in the LuPSMA group (p = 0.014) and between quartiles 2 and 3 in other SOC group (p = 0.037).

As an exploratory step, the cohort was divided into two groups based on a cut-off value of 200 ml of FDG-VOL which is based on literature [3]. Patients with FDG-VOL ≥ 200 ml had a shorter OS than patients with < 200 ml, regardless of the therapy received (5.5 months [95%CI 4.2-6.8] vs. 13.1 months [95%CI 11.7–14.4], p < 0.001).

### *PSA decline (*> *50%)*

**Fig. 5:**
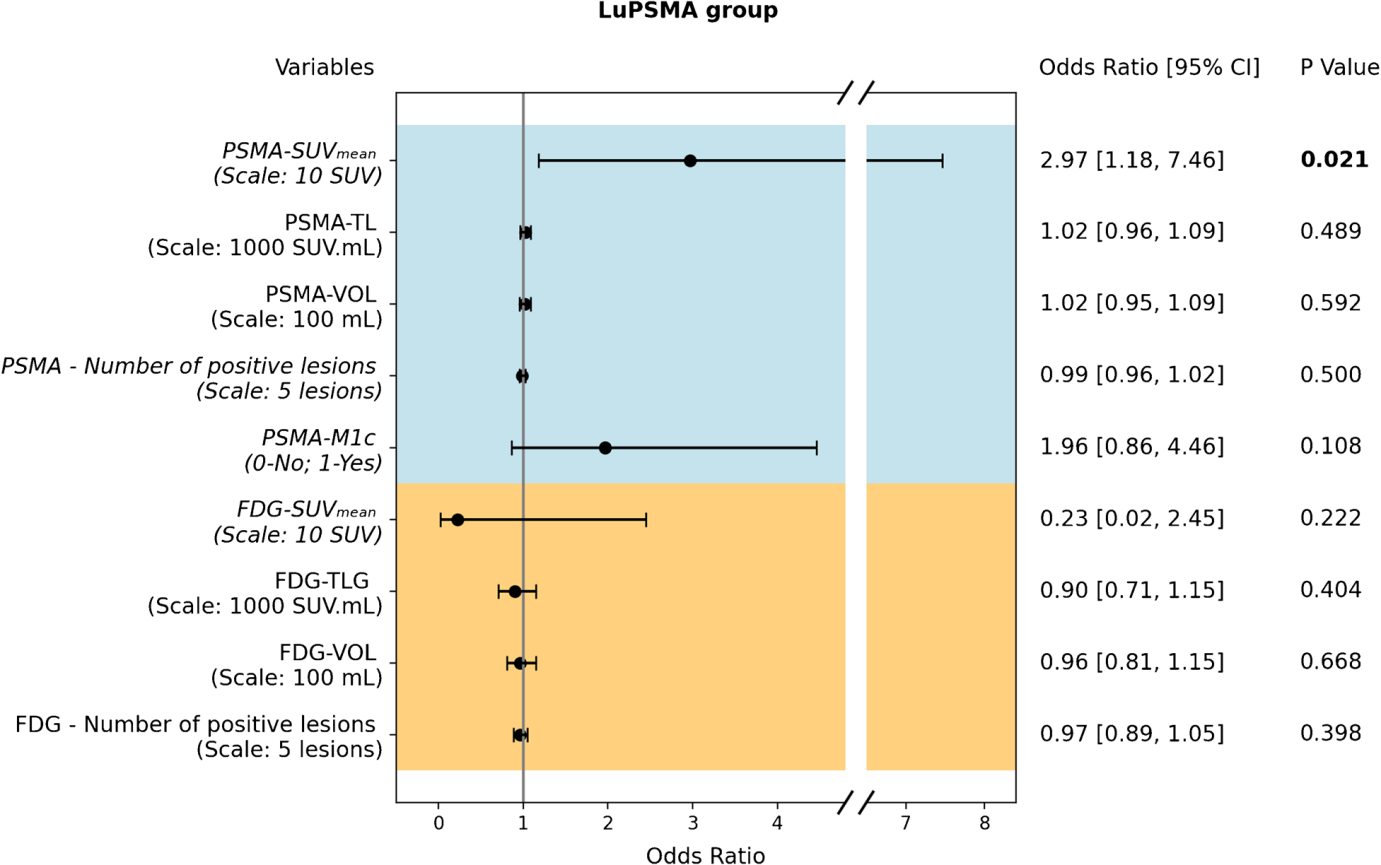
Forest plot of univariate logistic regression odds ratios for PSA Decline > 50% in patients undergoing LuPSMA therapy. Odds ratio, 95% confidence intervals (95% CI) as well as p-values of each univariate logistic regression model are presented

Figure 5 demonstrates the association of PET parameters with biochemical response (PSA decline > 50%). Only PSMA-SUVmean (OR 2.97 [95%CI 1.27, 8.16]; p = 0.021) was a significant predictor of PSA decline (> 50%). In Supplementary results, PSA decline logistic regression results are shown separately for radiotracers, [⁶⁸Ga]Ga-PSMA-11 versus [^18^F]-PSMA-1007.

### FDG-PSMA correlations

Spearman’s test showed no significant correlation (p = 0.118) between PSMA-SUVmean and FDG-SUVmean in patients treated with LuPSMA (Supplementary Fig. 3). Fourteen patients with non-measurable FDG-SUVmean were excluded from this analysis.

## Discussion

In this retrospective study, we demonstrate that both PSMA-PET and [^18^F]FDG-PET-derived molecular tumor volumes (PSMA-VOL and FDG-VOL) were significant negative prognosticators of survival in patients undergoing LuPSMA therapy. However, in multivariate analysis, only FDG-VOL remained significant. On the contrary, [^18^F]FDG-PET-derived metrics were not predictive of PSA response in patients treated with LuPSMA, while the average PSMA uptake (PSMA-SUVmean) was. These results confirm the prognostic value of FDG-VOL in patients with advanced prostate cancer undergoing both LuPSMA therapy and other SOC, while PSMA-SUVmean has predictive value specifically under LuPSMA therapy. Interestingly, a PSMA-PET derived parameter (PSMA-TL) is also prognostic in multivariable regression for patients not receiving LuPSMA therapy, highlighting the prognostic value of PSMA-PET also for other SOC options.

PSMA-PET is a routine procedure used to screen patients for LuPSMA therapy [16–18]. It has been shown that patients with low PSMA expression demonstrate unfavorable outcomes and that average PSMA expression is predictive of response to therapy [17, 19]. Therefore, the prospective VISION trial required patients to demonstrate uptake greater than the liver in all relevant metastases, without the presence of PSMA-negative disease, which was assessed by contrast-enhanced CT [9]. However, other trials have required more rigorous PSMA-PET criteria. The TheraP trial requested an SUVmax on PSMA-PET of at least 20 at a site of disease and greater than 10 at all other measurable metastases, and no presence of metastatic disease with discordant, i.e. 2-[^1^⁸F]FDG-positive and PSMA-negative findings [20]. More stringent selection likely resulted in higher PSA response rates, which were achieved in 65% in TheraP versus 46% in VISION [9, 20]. This is in line with other previous studies, as patients with a PSMA-SUVmean greater than 10 demonstrated a higher likelihood of PSA response as well as longer overall survival [3, 21]. Gafita et al. previously developed a nomogram to predict outcomes after LuPSMA that combines different clinical and imaging-related parameters including tumor PSMA-SUVmean on [^68^ Ga]Ga-PSMA-11 PET/CT [4]. In line with previous studies, we confirm the independent predictive value of PSMA-SUVmean on [^68^ Ga]Ga-PSMA-11 PET/CT for biochemical response along with its prognostic value in the univariate analysis in patients who underwent LuPSMA therapy. Interestingly, prognostication by PSMA-uptake varied by type of the PET radioligand used for imaging. In Supplementary Figs. 1 and 2, we show that the PSMA-SUVmean on [⁶⁸Ga]Ga-PSMA-11 PET was prognostic for overall survival, whereas the uptake on [^18^F]PSMA-1007 was not. The small cohort of patients imaged with [^18^F]PSMA-1007 (n = 45) might also have introduced a sampling bias, which could limit the applicability of the findings to different PET tracers in the setting of LuPSMA. In addition to the previously mentioned PSMA-PET exclusion criteria, the TheraP trial excluded patients with FDG-positive, PSMA-negative disease [5], given that PSMA-negative disease is not sufficiently targeted by LuPSMA therapy and may represent aggressive clones of prostate cancer, which might have undergone dedifferentiation or neuroendocrine transdifferentiation [19, 22, 23]. In contrast, the VISION trial employed PSMA-PET as the sole imaging criterion for therapy and did not evaluate patients with additional [^18^F]FDG-PET/CT [9]. The clinical relevance and necessity of [^18^F]FDG-PET imaging in addition to PSMA-PET remain subjects of debate. In the present study, we observed that patients with clinically significant FDG-PSMA mismatch findings had shorter OS compared to those without, irrespective of the received therapy (4.5 months vs. 10.6 months). This suggests that [^18^F]FDG-PET could be considered for assessing patient risk and determine the treatment intensity. Recently, Demirci et al. reported on TheraP-ineligible patients; of these, 77.5% were found to have FDG-PSMA mismatch disease. These had a lower PSA50 rate, an increased risk of PSA progression and a shorter OS than the TheraP-eligible group [24]. Moreover, the most recent findings of the TheraP study indicated that FDG-VOL greater than 200 ml was associated with lower biochemical responses and shorter progression-free survival regardless of randomly assigned therapy [3]. In line, in our study FDG-VOL was the only independent prognosticator of poor survival in patients both treated with LuPSMA therapy and those treated with other SOC. After dividing the present cohort into two groups at a cut-off value of 200 ml FDG-VOL, as suggested in several articles [3, 21], patients with FDG-VOL ≥ 200 ml had shorter OS than patients with < 200 ml (13.1 months vs. 5.5 months).

Certain findings such as PSMA-negative and FDG-positive bone lesions or bone marrow infiltration may be missed by PSMA-PET and CT imaging alone, however, the clinical relevance of this mismatch is yet unknown. Detection of PSMA-negative liver metastases of prostate cancer is improved by FDG-PET/CT and associated with short survival [25]. However, the overall diagnostic impact of dual PET/CT is limited: A recent analysis revealed that less than 5% of patients with FDG-positive but PSMA-negative findings are missed by only using PSMA and CT imaging [26].

The utility of [^18^F]FDG-PET extends beyond mismatch assessment. [^18^F]FDG-PET may serve as a biomarker of dedifferentiation and may assist in the selection and/or guidance of treatment. In line with previous research by Buteau and Ferdinandus et al. (3, 18), [^18^F]FDG-PET is a strong prognosticator. FDG-VOL was the only parameter independently associated with overall survival in both LuPSMA and other SOC patients. Specifically, mCRPC patients with [^18^F]FDG-PET tumor volume of 100 ml or higher should be prospectively evaluated for intensified treatment protocols, such as LuPSMA activity escalation, short interval dosing, or combination therapy. For example, Seifert et al. reported favorable disease control and overall survival in patients with liver metastases treated with LuPSMA and SIRT combination therapy [25].

This study comes with limitations. Analyses were conducted retrospectively and are therefore prone to selection bias. In the present study, some patients had baseline [⁶⁸Ga]Ga-PSMA-11 (n = 107) and others [^18^F]PSMA-1007 (n = 45). This may introduce heterogeneity in the study; however, the separate [⁶⁸Ga]Ga-PSMA-11 vs. [^18^F]PSMA-1007 analyses are aligned. Moreover, patients eligible for PSMA therapy and those excluded show distinct tumor phenotypes and findings for PSMA- and [^18^F]FDG-PET might not be generalizable to other mCRPC populations.

## Conclusion

In candidates for LuPSMA, several baseline PSMA-PET and [^18^F]FDG-PET metrics are prognostic. [^18^F]FDG-PET total tumor volume was independently associated with overall survival, irrespective of subsequent treatment decision. PSMA-PET SUVmean was associated with biochemical response to LuPSMA. Dual tracer imaging should further be assessed in prospective trials for mCRPC risk stratification and treatment guidance.

## Supplementary Information

Below is the link to the electronic supplementary material.

Supplementary Material 1 (DOCX 917 KB)

## Data Availability

The datasets generated during and/or analysed during the current study are available from the corresponding author on reasonable request.
