# Supplementary material for: Prognostic value of [18F]FDG- and PSMA-PET in patients evaluated for [177Lu]Lu-PSMA therapy of mCRPC
Source: Eur J Nucl Med Mol Imaging. 2025 Mar 21;52(9):3199–210. doi: 10.1007/s00259-025-07198-y (PMC12222416; doi:10.1007/s00259-025-07198-y)
Supplement: Supplementary file 1 — Supplementary Material 1 (DOCX 917 KB) [file 259_2025_7198_MOESM1_ESM.docx]

**Supplementary Data**

**Supplementary Tables and Figures**

| **Visual Assessment** | | |
| --- | --- | --- |
| **Assessment** | **PSMA-PET/CT** | **[^18^F]FDG-PET/CT** |
| **TNM staging** | **PROMISE criteria** | **Adapted PROMISE criteria** |
| Intensity of tracer uptake | ≥ Salivary glands = high | ≥ Liver x2 = high |
|  | ≥ Liver/spleen*= intermediate | ≥ Liver = intermediate |
|  | < Liver/spleen, > blood pool = low | < Liver/spleen, > blood pool = low |
| [^18^F]FDG-PSMA mismatch evaluation | Clinically relevant mismatch:  1) Visceral metastases/soft tissue lesions with longest diameter of ≥10 mm and PSMA uptake lower than liver  2) Lymph nodes with short axis diameter exceeds 15 mm and PSMA uptake lower than liver  3) ≥3 bone metastases with [^18^F]FDG uptake higher than liver and PSMA uptake lower than liver | |
|  | Match: All the lesions should have PSMA uptake higher than liver, and similar or lower [^18^F]FDG uptake | |
| **Semiquantitative Assessment** | | |
| **Assessment** | **PSMA-PET/CT** | **[^18^F]FDG-PET/CT** |
| Threshold SUV for volume segmentation | According to RECIP 1.0 | According to PERCIST |
|  | Higher than 3 for bone lesions and 4.3x(SUVmean+SD of liver))/SUVmean of liver for other regions | SUV higher than 1.5x liver SUVmean+2SD of liver SUV |
| Obtained Parameters from whole body tumor burden | PSMA-VOL, PSMA-TL, PSMA-SUVmean | FDG-VOL, FDG-TLG, FDG-SUVmean |
|  | Average SUVpeak of all PSMA-positive lesions, averageSUVmax of all PSMA-positive lesions | Average SUVpeak of all FDG-positive lesions, average SUVmax of all FDG-positive lesions |
|  | Number of PSMA-positive lesions | Number of FDG-positive lesions |
| Per tumor site evaluation (T, N, M1a, M1b, M1c) | PSMA-VOL, PSMA-TL per region | FDG-VOL, FDG-TLG per region |
| [^18^F]FDG-PSMA mismatch evaluation | According to PERCIST | |
|  | FDG-VOL mismatch, FDG-TLG mismatch | |

**Supplementary Table 1.** **Summary of Imaging Analyses.** *spleen was used for [^18^F]PSMA-1007

| Variable | Hazard Ratio [95% CI] | P-value |
| --- | --- | --- |
| PSMA_min_SUVmax | 0.548 [0.126, 2.377] | 0.422 |
| PSMA_max_SUVmax | 0.987 [0.94, 1.037] | 0.608 |
| PSMA_mean_SUVmax | 0.855 [0.669, 1.093] | 0.211 |
| PSMA_mean_SUVpeak | 0.681 [0.424, 1.095] | 0.113 |
| PSMA_mean_SUVmean | 0.557 [0.284, 1.095] | 0.09 |
| PSMA_TL_local_pelvic_lymph_nodes | 1.178 [0.885, 1.568] | 0.263 |
| PSMA_TL_M1a | 0.8 [0.626, 1.022] | 0.074 |
| PSMA_TL_M1b | 1.054 [1.022, 1.086] | **0.001** |
| PSMA_TL_M1c | 1.094 [0.865, 1.383] | 0.453 |
| PSMA_TV_local_pelvic_lymph_nodes | 1.93 [1.025, 3.633] | 0.042 |
| PSMA_TV_M1a | 0.749 [0.492, 1.141] | 0.178 |
| PSMA_TV_M1b | 1.057 [1.027, 1.089] | **<0.001** |
| PSMA_TV_M1c | 1.421 [1.033, 1.953] | **0.031** |
| PSMA_stage_M1a | 0.995 [0.638, 1.551] | 0.982 |
| PSMA_stage_M1b | 6.477 [0.9, 46.583] | 0.063 |
| FDG_min_SUVmax | 1.034 [0.574, 1.862] | 0.911 |
| FDG_max_SUVmax | 1.139 [1.01, 1.284] | **0.034** |
| FDG_mean_SUVmax | 1.109 [0.807, 1.525] | 0.522 |
| FDG_mean_SUVpeak | 1.131 [0.646, 1.981] | 0.667 |
| FDG_mean_SUVmean | 1.391 [0.637, 3.037] | 0.407 |
| FDG_TLG_local_pelvic_lymph_nodes | 1.456 [0.916, 2.315] | 0.112 |
| FDG_TLG_M1a | 0.846 [0.592, 1.209] | 0.359 |
| FDG_TLG_M1b | 1.223 [1.089, 1.373] | **0.001** |
| FDG_TLG_M1c | 1.79 [1.377, 2.327] | **<0.001** |
| FDG_MTV_local_pelvic_lymph_nodes | 6.008 [2.784, 12.961] | **<0.001** |
| FDG_MTV_M1a | 0.824 [0.546, 1.243] | 0.356 |
| FDG_MTV_M1b | 1.193 [1.098, 1.296] | **<0.001** |
| FDG_MTV_M1c | 1.417 [1.222, 1.643] | **<0.001** |
| FDG_stage_M1a | 0.966 [0.632, 1.476] | 0.873 |
| FDG_stage_M1b | 7.162 [1.752, 29.279] | **0.006** |
| FDG_stage_M1c | 1.067 [0.679, 1.676] | 0.78 |

**Supplementary Table 2.** **Results of univariate Cox Proportional Hazards Model of Overall Survival for aditional parameters within the LuPSMA group.** Hazard ratios (HR), 95% confidence intervals (95%CI) and p-values are presented for the LuPSMA treated and SOC group

| Variable | Hazard Ratio [95% CI] | P-value |
| --- | --- | --- |
| PSMA_min_SUVmax | 0.846 [0.198, 3.614] | 0.821 |
| PSMA_max_SUVmax | 1.022 [0.95, 1.1] | 0.561 |
| PSMA_mean_SUVmax | 1.099 [0.839, 1.441] | 0.493 |
| PSMA_mean_SUVpeak | 1.244 [0.762, 2.032] | 0.383 |
| PSMA_mean_SUVmean | 1.073 [0.723, 1.592] | 0.728 |
| PSMA_TL_local_pelvic_lymph_nodes | 1.071 [0.942, 1.218] | 0.296 |
| PSMA_TL_M1a | 1.132 [0.863, 1.484] | 0.37 |
| PSMA_TL_M1b | 1.059 [1.0, 1.121] | 0.05 |
| PSMA_TL_M1c | 1.172 [1.081, 1.27] | **<0.001** |
| PSMA_TV_local_pelvic_lymph_nodes | 1.269 [0.96, 1.678] | 0.094 |
| PSMA_TV_M1a | 2.409 [0.989, 5.868] | 0.053 |
| PSMA_TV_M1b | 1.059 [1.007, 1.114] | **0.027** |
| PSMA_TV_M1c | 1.54 [1.281, 1.851] | **<0.001** |
| FDG_min_SUVmax | 1.61 [0.374, 6.918] | 0.522 |
| FDG_max_SUVmax | 1.398 [1.157, 1.689] | **0.001** |
| FDG_mean_SUVmax | 1.769 [1.11, 2.82] | **0.016** |
| FDG_mean_SUVpeak | 2.843 [1.063, 7.605] | **0.037** |
| FDG_mean_SUVmean | 5.397 [1.103, 26.411] | **0.037** |
| FDG_TLG_local_pelvic_lymph_nodes | 1.341 [0.845, 2.126] | 0.213 |
| FDG_TLG_M1a | 1.908 [1.231, 2.958] | 0.004 |
| FDG_TLG_M1b | 1.089 [0.977, 1.213] | 0.124 |
| FDG_TLG_M1c | 1.129 [1.049, 1.216] | **0.001** |
| FDG_MTV_local_pelvic_lymph_nodes | 1.368 [0.909, 2.059] | 0.133 |
| FDG_MTV_M1a | 1.561 [1.166, 2.091] | **0.003** |
| FDG_MTV_M1b | 1.081 [0.987, 1.185] | 0.094 |
| FDG_MTV_M1c | 1.121 [1.053, 1.193] | **<0.001** |
| FDG_stage_M1a | 1.774 [0.97, 3.243] | 0.063 |
| FDG_stage_M1b | 1.239 [0.442, 3.477] | 0.684 |
| FDG_stage_M1c | 2.263 [1.207, 4.243] | **0.011** |

**Supplementary Table 3.** **Results of univariate Cox Proportional Hazards Model of overall survival for aditional parameters of SOC group.** Hazard ratios (HR), 95% confidence intervals (95%CI) and p-values are presented for the LuPSMA treated and SOC group

**
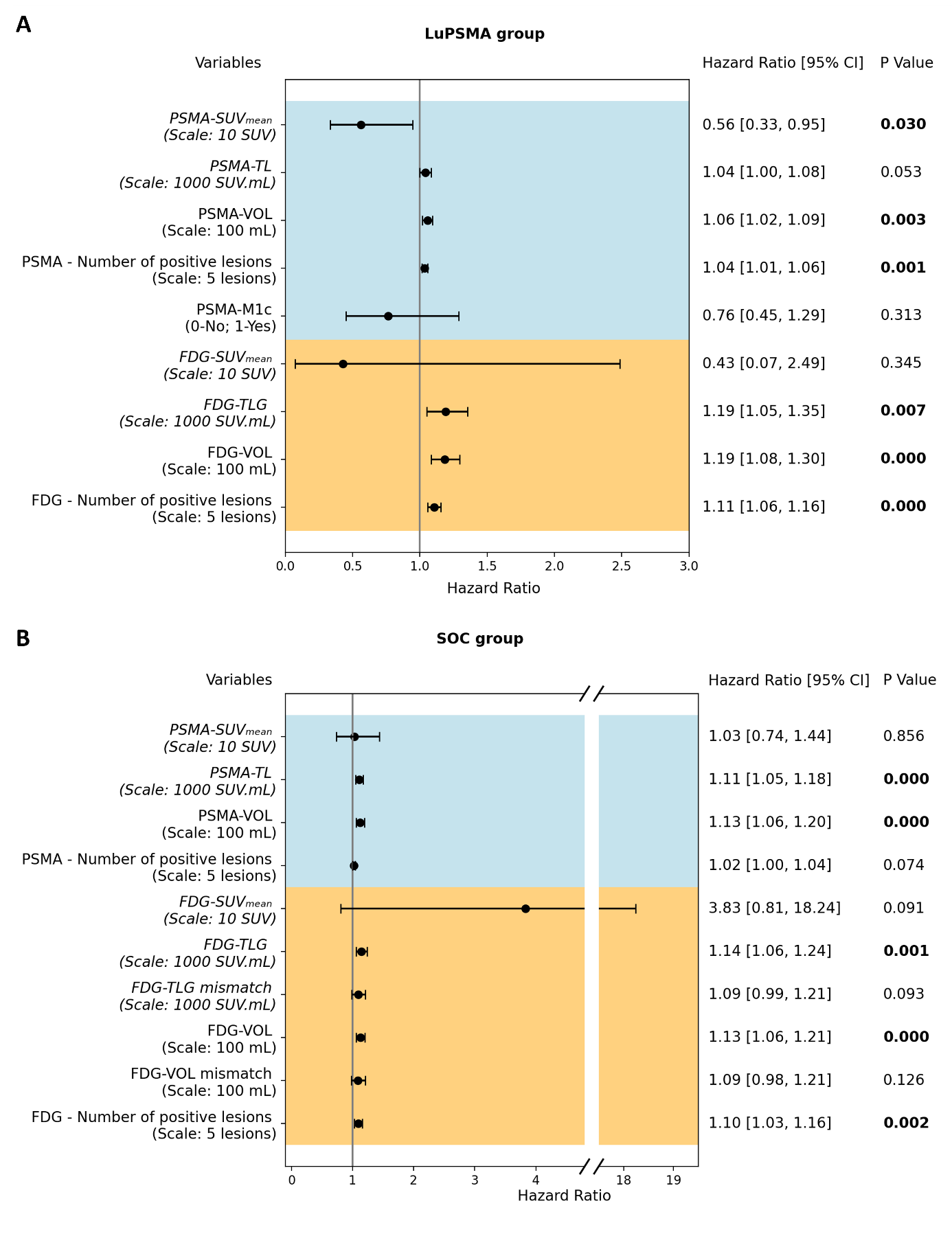
**

**Supplementary Figure 1. Forest plots of univariate Cox Proportional Hazards Model of [⁶⁸Ga]Ga-PSMA-11** **group in A) LuPSMA and B) other SOC group.** Hazard ratios, 95% confidence intervals (95% CI) as well as p-values of each univariate cox model are presented.

**
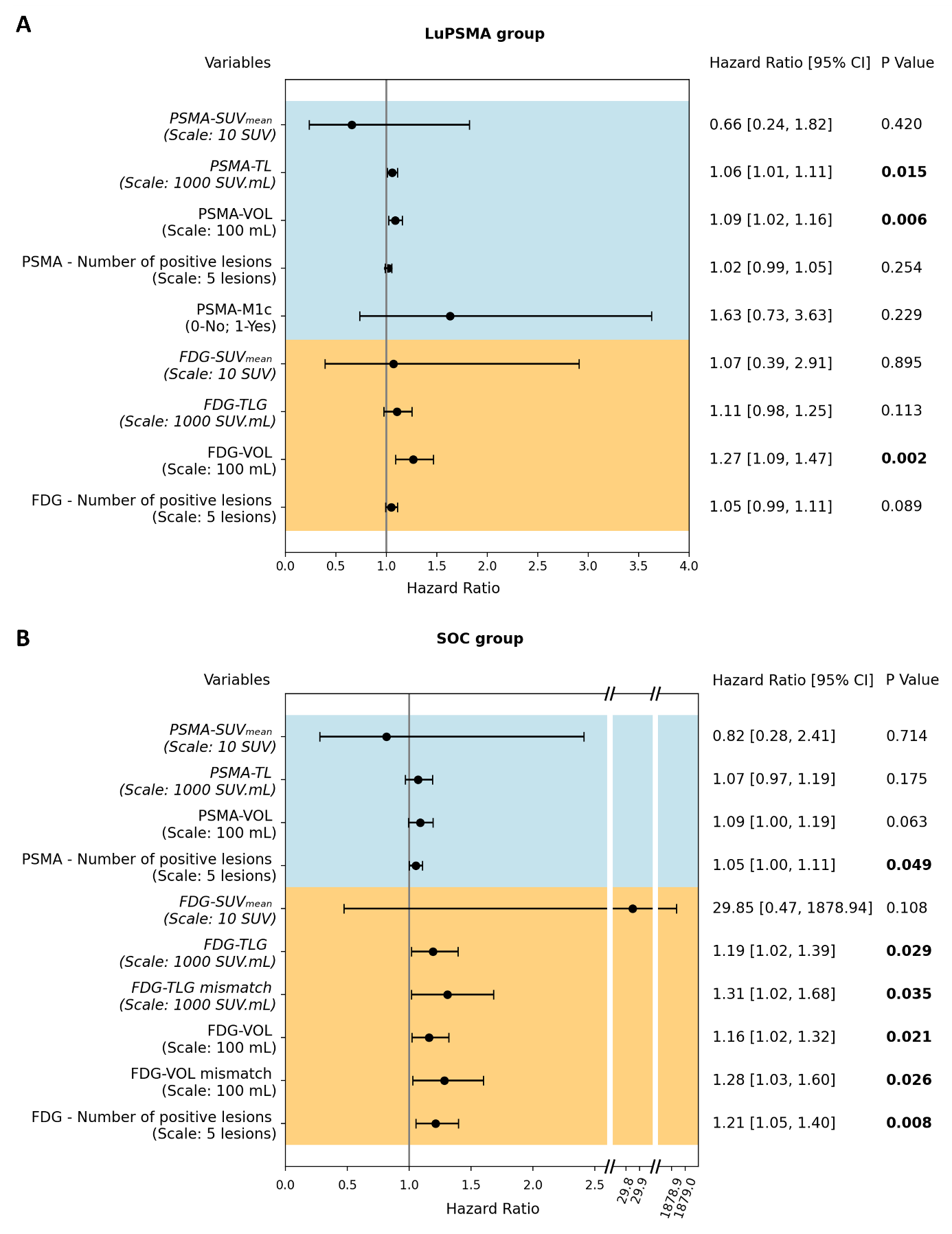
**

**Supplementary Figure 2. Forest plots of univariate Cox Proportional Hazards Model of [^18^F]PSMA-1007** **group in A) LuPSMA and B) SOC group.** Hazard ratios, 95% confidence intervals (95% CI) as well as p-values of each univariate cox model are presented.


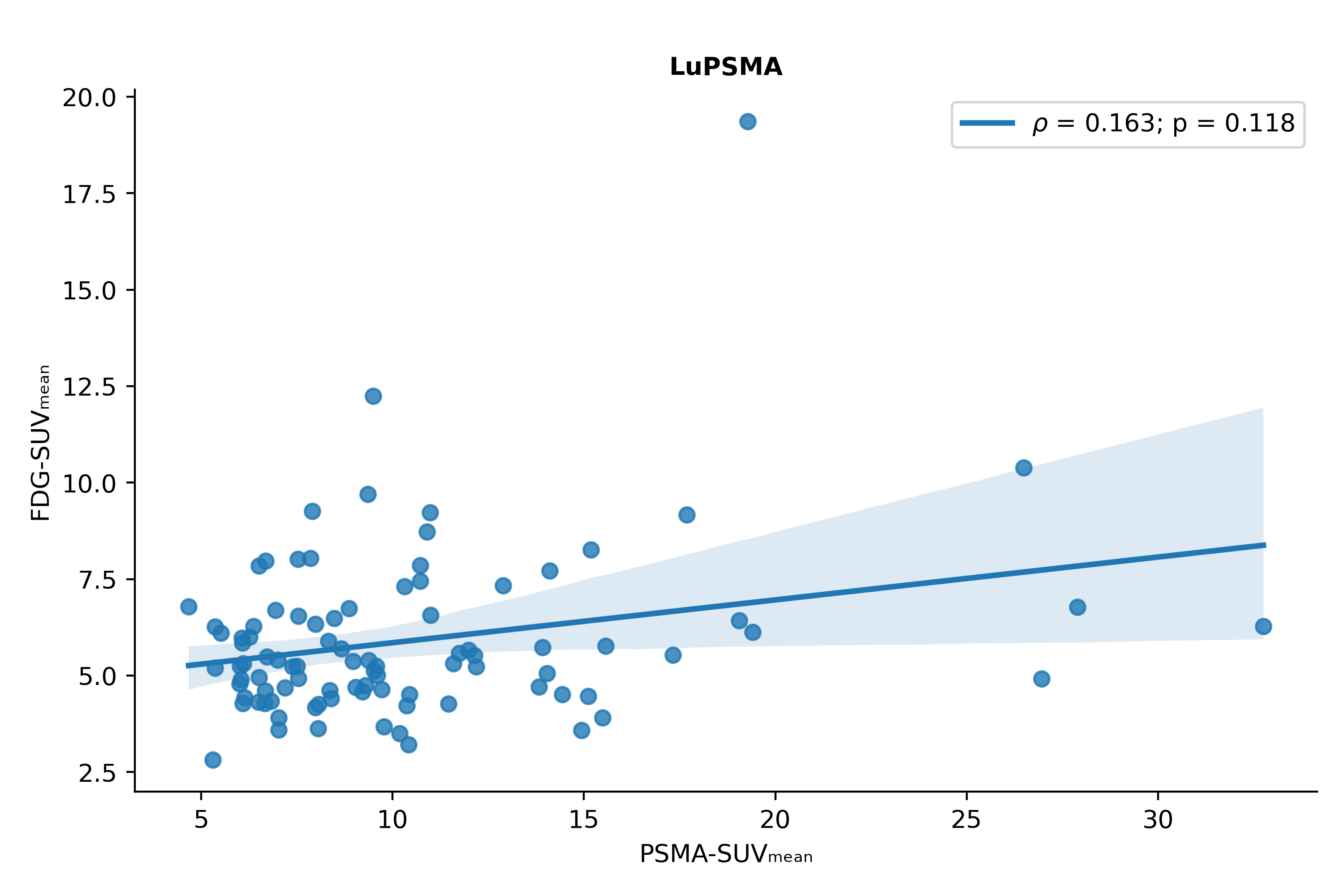


**Supplementary Figure 3. Correlation between FDG mean SUV and PSMA mean SUV.** Correlation was assessed by Spearman’s test, and correlation coefficient and p-value are shown.

**Supplementary Results**

**Overall Survival Evaluation in [⁶⁸Ga]Ga-PSMA-11 PET and [^18^F]PSMA-1007 PET groups**

**[⁶⁸Ga]Ga-PSMA-11 PET/CT:** The univariate cox model results for the [⁶⁸Ga]Ga-PSMA-11 group are presented in Supplementary Figure 1. In LuPSMA patients, all assessed PET parameters except PSMA-TL, PSMA-M1c and FDG-SUVmean were significant prognostic factors of overall survival. PSMA-SUVmean (HR 0.56 [95%CI 0.33 - 0.95]; p=0.030) was a protective significant prognostic factor and PSMA-VOL (HR 1.06 [95%CI 1.02 - 1.09]; p=0.003), number of PSMA-positive lesions (HR 1.04 [95%CI 1.01 - 1.06]; p=0.001) were significantly prognostic of worse outcome. Regarding FDG paramenters, FDG-TLG (HR 1.19 [95%CI 1.05 - 1.35]; p=0.007), FDG-VOL (HR 1.19 [95%CI 1.08 - 1.30]; p<0.001) and number of FDG-positive lesions (HR 1.11 [95%CI 1.06 - 1.16]; p<0.001) were significantly prognostic of worse outcome.

In SOC patients, PSMA-TL (HR = 1.11 [1.05 - 1.18]; p<0.001) and PSMA-VOL (HR 1.13 [95%CI 1.06 - 1.20]; p<0.001), were significantly prognostic of worse outcome. For [^18^F]FDG-PET, FDG-TLG (HR 1.14 [95%CI 1.06 - 1.24]; p=0.001), FDG-VOL (HR 1.13 [95%CI 1.06 - 1.21]; p<0.001), and number of FDG-positive lesions (HR 1.10 [95%CI 1.03 - 1.16]; p=0.002) were significantly prognostic of worse outcome.

**[^18^F]PSMA-1007 PET:** The univariate cox model results for the [^18^F]PSMA-1007 group are presented in Supplementary Figure 2. In LuPSMA patients, only PSMA-TL (HR 1.06 [95%CI 1.01 - 1.11]; p=0.015) and PSMA-VOL (HR 1.09 [95%CI 1.02 - 1.16]; p=0.006) were significantly prognostic of worse outcome. Regarding [^18^F]FDG-PET paramenters, only FDG-VOL (HR 1.27 [95%CI 1.09 - 1.47]; p=0.002) was significantly prognostic of worse outcome.

In SOC patients, number of PSMA-positive lesions (HR 1.05 [95%CI 1.00 - 1.11]; p=0.049) was significantly prognostic of worse outcome. For [^18^F]FDG-PET, FDG-TLG (HR 1.19 [95%CI 1.02 - 1.39]; p=0.029), FDG-TLG mismatch (HR 1.31 [95%CI 1.02 - 1.68]; p=0.035), FDG-VOL (HR 1.16 [95%CI 1.02 - 1.32]; p=0.021), FDG-VOL mismatch (HR 1.28 [95%CI 1.03 - 1.60]; p=0.026), and number of FDG-positive lesions (HR 1.21 [95%CI 1.05 - 1.40]; p=0.008) were significantly prognostic of worse outcome.

**PSA decline (>50%) in [⁶⁸Ga]Ga-PSMA-11 PET and [^18^F]PSMA-1007 PET groups**

In [⁶⁸Ga]Ga-PSMA-11 group, only PSMA-SUVmean (OR 3.16 [95%CI 1.20 – 10.76], p=0.030) was a significant predictor of PSA decline (>50%). In [^18^F]PSMA-1007 group no significant predictors were found.
